# Implementation of Unobtrusive Sensing Systems for Older Adult Care: Scoping Review

**DOI:** 10.2196/27862

**Published:** 2021-10-06

**Authors:** Nikita Sharma, Jeroen Klein Brinke, J E W C Van Gemert-Pijnen, L M A Braakman-Jansen

**Affiliations:** 1 Centre for eHealth and Wellbeing Research, Department of Psychology, Health & Technology University of Twente Enschede Netherlands; 2 Pervasive Systems, Faculty of Electrical Engineering, Mathematics and Computer Science University of Twente Enschede Netherlands

**Keywords:** elderly care, unobtrusive, sensing system, caregiving, implementation, mobile phone, older adults

## Abstract

**Background:**

The continuous growth of the older adult population will have implications for the organization of health and social care. Potentially, in-home monitoring unobtrusive sensing systems (USSs) can be used to support formal or informal caregivers of older adults, as they can monitor deviant physical and physiological behavior changes. Most existing USSs are not specific to older adult care. Hence, to facilitate the implementation of existing USSs in older adult care, it is important to know which USSs would be more suitable for older adults.

**Objective:**

This scoping review aims to examine the literature to identify current USSs for monitoring human activities and behaviors and assess their implementation readiness for older adult care.

**Methods:**

We conducted a structured search in the *Scopus*, *Web of Science*, and *ACM Digital Library* databases. Predefined inclusion criteria included studies on unobtrusive sensor-based technology; experimental in nature; aimed at monitoring human social, emotional, physical, and physiological behavior; having the potential to be scalable in in-home care; and having at least 5 adults as participants. Using these criteria, we screened studies by title, abstract, and full text. A deductive thematic analysis based on the Proctor implementation framework along with an additional outcome of *external validity* was applied to the included studies to identify the factors contributing to successful implementation. Finally, the identified factors were used to report the implementation readiness of the included studies for older adult care.

**Results:**

In this review, 52 studies were included. Deductive analysis using the implementation framework by Proctor resulted in six factors that can contribute to the successful implementation of USSs in older adult care: study settings, age of participants, activities monitored, sensor setup, sensing technology used, and usefulness of USSs. These factors were associated with the implementation outcomes as follows: study settings and age of participants contributed to external validity, sensor setup contributed to acceptability, usefulness of USSs contributed to adoption, activities monitored contributed to appropriateness, and sensing technology used contributed to implementation cost. Furthermore, the implementation assessment of the included 52 studies showed that none of the studies addressed all the identified factors. This assessment was useful in highlighting studies that have addressed multiple factors; thus, these studies represent a step ahead in the implementation process.

**Conclusions:**

This review is the first to scope state-of-the-art USSs suitable for older adult care. Although the included 52 USS studies fulfilled the basic criteria to be suitable for older adult care, systems leveraging radio frequency technology in a no-contact sensor setup for monitoring life risk or health wellness activities are more suitable for older adult care. Finally, this review has extended the discussion about *unobtrusiveness* as *a property of systems that cannot be measured in binary because it varies greatly with user perception and context*.

## Introduction

### Background

The older adult population has been increasing at an alarming rate over the past few years. According to the United Nations World Population Prospect Report, the population of people aged ≥65 years will approximately double, rising from 9% in 2019 to 16% in 2050. Consequently, by 2050, 1 in 4 persons will be aged ≥65 years in Europe and Northern America [1]. This anticipated growth of the older adult population will have a direct impact on the economy, employment, social care, and health care services worldwide [2,3]. With increasing age, older adults become more prone to fatal diseases, mandating continuous care by formal (trained professionals) or informal (family, friends, and relatives) caregivers. Most older adults prefer to stay in their own homes, which increases the burden on informal caregivers [4]. Owing to this, detrimental effects on the physical, emotional, and social well-being of caregivers have been observed [5,6]. Thus, to provide continuous care without burdening informal caregivers and adhering to the needs of older adults, intelligent in-home monitoring technological solutions are proposed and demanded [7-9].

Many in-home monitoring technological solutions that can recognize various physical and physiological human activities have been designed and evaluated. The most common human activity recognition (HAR) solutions include (1) wearable sensing systems (eg, smartwatches, smart clothing, and mobile phones), (2) vision-based systems (eg, surveillance cameras and Kinect), and (3) radio frequency (RF)–based sensing systems (eg, Wi-Fi, radar, and wireless sensors embedded in daily-use objects). The aforementioned solutions have the potential to assist caregivers, but most of them are not favorable for older adult care. *Wearable sensing systems* have acceptability issues because wearing monitoring devices all the time leads to feelings of stigmatization in older adults [10]. Wearables also have feasibility issues when used by older adults with cognitive impairments as they might forget to wear them [11]. *Vision-based systems* require users to be in their line of sight (LOS) and are consequently prone to privacy and ethical issues [12]. *RF-based systems* overcome the disadvantages of wearables and vision-based systems [13]. Potentially, RF systems could be considered more privacy aware than vision-based systems, as the raw data are not easily interpretable by humans and require complex data processing. Most importantly, they are *unobtrusive*, such that the user does not have to wear the device (device-free sensing) for continuous monitoring and can operate in a non-LOS (NLOS) region, thus making them more suitable for older adult care [4].

With the advancement in technology, the meaning of *unobtrusive* has evolved. Initially, wearables were labeled *unobtrusive* as they are noninvasive to the human body [14]. Currently, the possibility of using sensing systems far away from the human body (device free) for HAR is being explored and such systems are now referred to as unobtrusive systems [15]. This shift in the interpretation of *unobtrusive* as per convenience is because of the lack of a consensus definition or framework for unobtrusiveness. To eliminate the existing biases regarding the meaning of *unobtrusive*, the dictionary meaning was used in this paper. According to the dictionary, unobtrusive means “not noticeable or seeming to fit in well with the things around or something that does not draw attention” [16,17]. Evolving from this meaning, a sensor-based technology that does not draw the user’s attention or demand their direct involvement, while blending well with the surroundings, can be termed as an unobtrusive sensing technology (UST). The systems that leverage such technologies were considered as unobtrusive sensing systems (USSs) and included in this study. For example, in a study by Adib et al [15], radio wave sensors were used as a UST to determine the physiological activities (heart rate [HR] and breathing rate [BR]) of healthy human subjects. Similarly, Wi-Fi channel state information used to detect physical activities such as walking, sitting on a chair, and falling can be considered as UST [18]. In line with the aforementioned definition and the conceptual framework for obtrusiveness by Hensel et al [19], wearables, smartphones, camera-based systems, and any systems that require direct human contact are categorized as obtrusive sensing systems. It should be noted that unobtrusiveness does not account for the privacy and sustainability aspects (specifically for this study).

In the past few years, the focus of sensing research has shifted toward unobtrusive sensing specifically to support older adults, patients, and disabled persons. As a result, intelligent state-of-the-art USSs are being developed with the aim of supporting independent living by leveraging different USTs (RF identification, infrared [IR], and channel state information) for HAR and health monitoring. The European technology readiness level (TRL) scale can be used to measure the maturity and hence implementation possibilities of state-of-the-art USTs [20]. A few of the available USTs were translated to commercial products, such as AbiSensor [21] (TRL 7/9), or some are in real-life demonstration phases, such as the Gator Tech smart house, MavHome prototype, etc (TRL 5/7) [22,23], and can thus be seen as an initiative to use USSs in older adult care. Finally, most of the advanced technologies are still in the exploratory and validation phases. For example, radar-based systems were developed for monitoring activities of daily life and vital signs but tested in controlled laboratory settings with young adults (TRL 2/4) [15,24-29]. The available state-of-the-art UST research or prototype (TRL 2/4) can also be used to support older adult care, given their effective implementation process. In this regard, this study aims to bring forward exploratory technologies and systems in TRL 2/4, as they are not widely adopted by current health care organizations or older adult homes despite their possible benefits.

Ideally, a successful implementation process incorporates the user’s needs and perspectives (accounts for acceptability) [30], evaluates the technical maturity of systems (accounts for reliability) [31], and undertakes challenges faced by prospective industries or organizations (accounts for feasibility) [32,33]. Thus, with the development of UST, parallel research on its effective implementation is required [34]. To facilitate the implementation process in health care, frameworks such as NASSS (nonadoption, abandonment, scale-up, spread, sustainability) are popular and effective [35]. It can be used to preassess the technology for implementation after its development. However, to make the development implementation aware, the psychometric and pragmatic implementation constructs or outcomes that serve as preconditions for achieving intended results or changes should be considered from the development phase itself [36]. For this, basic frameworks such as the one by Proctor et al [37] can be used. This framework uses eight distinct implementation outcomes—acceptability, adoption, appropriateness, feasibility, ﬁdelity, implementation cost, penetration, and sustainability—encompassing the implementation process, success, and outcomes; hence, it could be used to make early-stage technologies, such as UST, implementation aware.

### Objectives

Along with the development of new technologies, existing state-of-the-art technologies could be made implementable to facilitate and accelerate the process of using USSs in older adult care. To achieve this, a consolidated overview of existing research on USSs followed by an evaluation of their implementation readiness is required. Therefore, first, this study aims to identify existing research or validation phase studies using USSs and their underlying technologies for monitoring physical, physiological, and emotional behavior changes or activities of human adults that are suitable for older adult care through a scoping review. Second, the study aims to evaluate them for implementation readiness using the framework by Proctor et al [37] for facilitating and accelerating their use in older adult care. In addition to the framework by Proctor et al [37], *external validity* was added as a relevant outcome, considering the novelty and technical nature of USSs [38].

## Methods

### Overview

As the technology is developing rapidly, a time-bound scoping review was conducted [39]. The review followed the five stages of the methodological framework for scoping reviews by Arksey and O’Malley [40]. These stages were (1) identifying the research question (Introduction section); (2) identifying relevant studies (*Identifying Relevant Studies* section); (3) selection of relevant studies (*Selection of Relevant Studies* section); (4) charting the data obtained from selected literature (*Data Extraction* section); and (5) collating, summarizing, and reporting the results (*Results* section). Two researchers were involved in the review process. The primary researcher (NS) was responsible for title, abstract, and full-text screening of the identified literature, followed by data extraction and manuscript writing. To ensure the quality of the review, the second reviewer (JKB) carried out 25% of full-text screening, followed by writing and evaluating parts of the *Results* sections.

### Identifying Relevant Studies

This review required technical literature with its application in social science. Therefore, three electronically available databases, Scopus, Web of Science, and ACM Digital Library, including papers from both engineering and social science fields, were explored. A search string for identifying existing USSs was formed. The search string was finalized after discussion with an information specialist from the Faculty of Behavioral, Management, and Social Sciences at the University of Twente. The search string was divided into five sets: type of system, type of technology, type of user, type of behavior or activity, and type of observation. The keywords used are presented in Table 1.

**Table 1:** Keywords for the search string.

Sets of keywords	Search words
Type of systems	*Unobtrusive*, *Nonintrusive*, *Non-wearable*, *Contactless*, *Wireless*
Type of technology	*Sensing technology*
Type of users	*Human adults* (this by default includes older adults)
Type of activity or behavior	*Social*, *Emotional*, *Physical*, *Physiological*
Type of observation	*Recognition*, *Detection*, *Monitoring*, *Tracking*

We found a total of 3157 research articles by using a search string composed of these keywords (Scopus: 1171; Web of Science: 1524; and ACM Digital Library: 462). The search strings are provided in Multimedia Appendix 1. The search included the title, keywords, and abstracts from January 2011 to March 2020 (last decade). The time span was limited, as we aimed to identify state-of-the-art USTs (time-bound scoping review). No other search limitations were imposed.

### Selection of Relevant Studies

The title, abstract, and full-text screening was conducted using the web-based software platform Covidence [41]. To systematically report the process of identified articles, the PRISMA (Preferred Reporting Items for Systematic Reviews and Meta-Analysis) guidelines extension for scoping reviews were used [42]. For the title, abstract, and full-text screening, the inclusion and exclusion criteria were defined considering the use of USSs in older adult care. Textbox 1 details the inclusion and exclusion criteria.

**Inclusion and exclusion criteria.**

**Inclusion criteria**

- Sensor-based technology but unobtrusive in nature
- Experimental studies demonstrating practical application of technology (including laboratory or field testing)
- Studies with the aim of monitoring, detecting, recognizing, or tracking human social, emotional, physical, and physiological behavior
- Studies that can be applied in in-home care or showing or possible applications in health care (such as monitoring vital signs)
- Studies with human adults as participants (≥18 years)

**Exclusion criteria**

**Figure 1:**
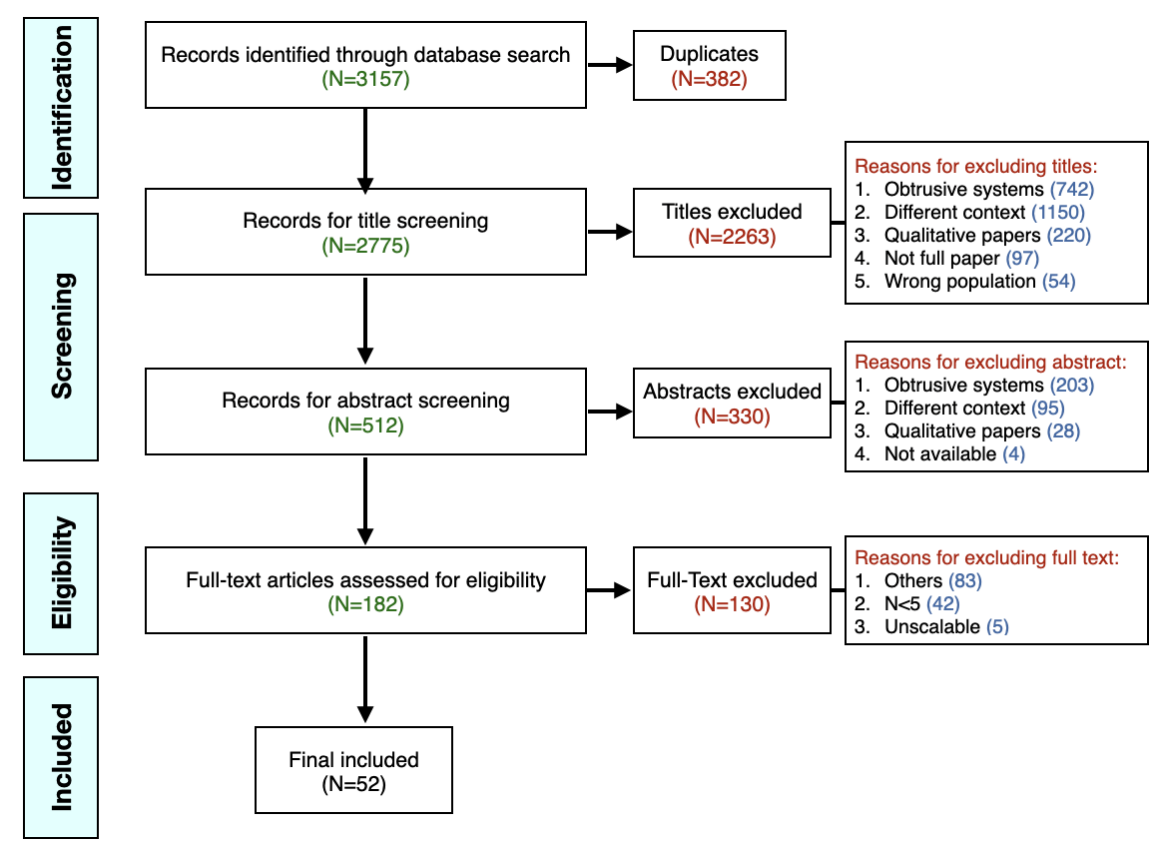
PRISMA (Preferred Reporting Items for Systematic Reviews and Meta-Analysis) flow diagram.

- Wearables, smart phone–based systems, and camera-based systems (labeled as *Obtrusive systems* in Figure 1)
- Review papers and qualitative studies (labeled as *qualitative studies* in Figure 1)
- Studies suggesting only algorithmic, hardware improvements and papers with different aims then desired (labeled as *Different Context* in Figure 1)
- Sensor-based technology that are used in a wide range of domains such as environment monitoring, driver behavior monitoring, etc (labeled as *Different Context* in Figure 1)
- Studies on infants and animals (labeled as *Wrong target group* in Figure 1)

First, from 3157 papers, 382 duplicate papers were removed, and the remaining 2775 unique papers were used for title screening. Using the aforementioned inclusion and exclusion criteria in title screening, 2263 studies were excluded, and 512 studies were selected for the abstract screening step where another 330 articles were excluded, resulting in 182 studies for full-text screening. Among the excluded 330 studies, most studies (n=203) used obtrusive sensing (mobile based or vision based) systems and 95 studies had different contexts than the aim of this review. For full-text screening of 182 studies, two additional inclusion and exclusion criteria were added by discussing with all the authors. These criteria aimed to filter studies with inadequate evidence for upscaling them in older adult care. The additional inclusion and exclusion criteria are detailed in Textbox 2.

**Additional inclusion and exclusion criteria.**

**Inclusion criteria**

- The performance of a system should be scalable and competent with state-of-the-art systems, that is, the accuracy or equivalent measure of the proposed system should be more than 80%.
- Number of participants should be greater or at least equal to 5 (N≥5).

**Exclusion criteria**

- Papers having low performance (or accuracies or other equivalent measure; labeled as *Unscalable* in Figure 1).
- Papers that have tested the systems with less than 5 participants (labeled as *N<5* in Figure 1).

Finally, out of 182 research articles, 52 articles were found relevant and were added in this scoping review upon agreement between reviewers NS (reviewed all 182 studies independently) and JKB (reviewed 46 studies independently). Studies with discrepancies were discussed until a consensus was reached. Interreviewer reliability was calculated using the Cohen κ coefficient. The Cohen κ for 25% of full-text articles was 0.81, which indicates almost perfect agreement between reviewers [43]. Among the excluded 130 studies, most were in the category of obtrusive systems followed by different contexts (both labeled as *Others* in Figure 1), participants less than five, and unscalable systems. Figure 1 (PRISMA flow diagram) illustrates the step-by-step flow of information through different phases of study selection. All of the aforementioned steps were continuously discussed and reported by the primary researcher with the research committee.

### Data Extraction

Descriptive analysis was used to chart the key information of the 52 USS studies, including general study description elements: study aim, study design, study settings, participant information, and the main results. In addition to general study description elements, technological description elements, including activity or behaviors monitored, sensor used, and data analysis methods, were also added. The obtained information is presented in Multimedia Appendix 2 [11,15,18,24-29,44-86].

The implementation outcomes defined in the framework by Proctor et al [37] were used to evaluate the implementation readiness of 52 identified USSs for older adult care (RQ2). The eight conceptually distinct implementation outcomes—acceptability, adoption, appropriateness, feasibility, fidelity, implementation cost, penetration or coverage, and sustainability—are helpful in understanding and conceptualizing the implementation process, success, and outcome. Considering that USSs are still in the developing stage, implementation outcomes that belong to early- to midstage implementation (acceptability, adoption, appropriateness, and implementation cost) were used [37]. Furthermore, an additional outcome, external validity, which can contribute to the early implementation stage was added [38]. These outcomes were translated according to the older adult care context (Textbox 3). Next, a deductive thematic analysis [87] based on the translation of the framework by Proctor et al [37] was performed on the 52 studies to identify factors (or themes) that can contribute to the successful implementation of USSs. Furthermore, textual analysis was performed within the identified themes to identify the subthemes. These key factors are elaborated as key themes in the *Results* section. Finally, the included studies were assessed on the basis of identified factors for implementation readiness. The Atlas.ti software (8.4.5) was used for deductive and textual analyses [88].

**Implementation outcomes and their translation for older adult care.**

**External validity**

- Meaning: it is the extent to which the results of a study can be generalized to and across other situations, people, stimuli, and times [38,89].
- Translation: for older adult care, a study should provide valid results in older adult homes and with older adults. Therefore, studies that were performed in appropriate settings (real-life settings) and with the intended age group of participants (older adults) have checked external validity [90].

**Acceptability**

- Meaning: the perception among stakeholders that an intervention is agreeable or fit with user’s expectations [37,91].
- Translation: for older adult care, the acceptance of any new technology is related to ease with which it can be integrated in their lifestyle, hence they require systems which provide them freedom without continuously bothering them [14,90]. Therefore, sensors which provide user freedom to roam around without wearing it or staying in the line of sight might have more chances of acceptance by older adults.

**Adoption**

- Meaning: the intention, initial decision, or action to try to use a new intervention [37,91].
- Translation: the process of adoption begins with the intention of research. In this review, more than 50% of included studies discussed the advantages of their USS for older adult care. This indicates the intention or possibility to use their intervention for older adult care.

**Appropriateness**

- Meaning: the perceived fit or relevance of the intervention in a particular setting or for a particular target audience or problem [37,91].
- Translation: in older adult care, activities monitored are of interest and value from the perspective of stakeholders such as formal or informal caregivers, older adults, and involved organizations (such as older adult homes and participating companies). Therefore, studies that monitor activities relevant to older adult care are more appropriate [9].

**Implementation cost**

- Meaning: it encapsulates cost of intervention, implementation strategy, and the location of service delivery [37,91].
- Translation: the cost of implementation involves the cost of systems, efforts, and time required to install the systems. Thus, to implement USSs in older adult care, studies using technologies that require minimum cost for deployment, maintenance can be considered [4].

## Results

### Overview

An overview of the included 52 studies is provided in Multimedia Appendix 2. From this, it can be observed that most of the research in the field of USSs was conducted in the last 5 years (44/52, 85% of studies were from 2015 to 2019). Moreover, the geographical locations of these existing research studies show that most of the studies were conducted in Asia (20 studies), followed by North America (19 studies), Europe (12 studies), and Australia (1 study).

From deductive thematic analysis based on implementation outcomes by Proctor et al [37], 6 key factors that can contribute to successful implementation were identified: *sensor setup*, *study settings, age of participants, type of activities monitored, sensing technology used, and usefulness of unobtrusive systems*. The implementation outcomes were associated with these factors as follows: study settings and age of participants contribute to external validity, sensor setup contributes to acceptability, usefulness of USSs contributes to adoption, activities monitored contribute to appropriateness, and sensing technology used contributes to implementation cost. The detailed explanation of factors, the associated implementation outcome, and the corresponding subthemes identified by textual analysis is elaborated in the *Key themes: Factors contributing to implementation* section. Finally, the results of the assessment of the identified studies for implementation readiness are presented.

### Key Themes: Factors Contributing to Implementation

#### Theme 1: Sensor Setup

##### Overview

The sensor setup can be referred to as an arrangement of sensors in the user’s surroundings. The studies included in this review were unobtrusive in nature. Within unobtrusive sensing, two broad patterns in the sensor setup were identified: (1) *no-contact sensor setup* and (2) *indirect contact sensor setup.* Table 2 lists the identified studies into these categories.

**Table 2:** Sensor setup.

Sensor setup: arrangement of sensors or sensing units			Included studies
**No-contact sensor setup**			
	One or a couple of sensors or sensing units placed at a reasonable distance from the user (~3-9 m) and operates in NLOS^a^ scenarios	[15,18,24-26,28,29,44,45,53,60,68,72,77,78,80,82]	
	One or a couple of sensors or sensing units placed close to the user (approximately 0.5-3 m) and evaluated in only LOS^b^ or close proximity scenarios	[11,27,50-52,56-59,62,64,65,69,71,75,76,79,81,83,85,86]	
	Sensors or sensing units placed in surroundings objects such as on doors, fridge, walls, among others	[46,48,55]	
**Indirect contact sensor setup**			
	One or a couple of sensors or sensing units embedded in daily-use objects such as mattress, chair, floor tiles, among others. Require user to be in indirect contact with respective objects	[47,49,54,61,63,66,67,70,73,74,84]	

^a^NLOS: non–line of sight.

^b^LOS: line of sight.

##### No-Contact Sensor Setup

It can be seen as a single sensing unit consisting of either a single sensor or an assembly of heterogeneous sensors capable of gathering a subject’s intended activity data from a distance. Within the no-contact sensing system, three patterns based on the number of sensors and the distance of operation were observed:

1. Sensor setups with only one or a couple of sensors or sensing units placed at a distance between 3 and 9 m and can operate in NLOS: systems that have this type of sensor setup are potentially unobtrusive because of their larger coverage and easy deployment as the device is compact in nature. Such systems can be placed in the corner of a house or room (almost unnoticeable), and desirable results can still be obtained. For example, the FMCW (frequency modulated continuous wave) radar was used to track an individual’s walking gestures beyond the wall or at approximately 9 m [44]. Among the 52 included studies, 17 were identified in this category.
2. Sensor setups with only one or a couple of sensors or sensing units but tested for smaller distances 0.5-3 m in LOS or close proximity: this category includes studies that were tested at a limited distance with the possibility of scaling up by evaluating them at larger distances. For example, a contactless sleep-sensing system was developed to continuously track sleep quality using commercial off-the-shelf radar modules [69]. The system was placed at a distance of 0.5 m from the user in the experiments. More experiments at larger distances or modifications in this system can be carried out to upscale the system. A total of 21 such studies were found.
3. Sensor setup with a number of sensors or sensing units mounted on surrounding objects at multiple locations and works only when the user is in LOS: this type of sensor setup enables close and accurate monitoring of individuals when in the LOS of the sensors. Higher accuracy makes these systems more reliable, but unobtrusiveness is compromised as they have to be mounted on multiple locations in close proximity to the user. They might face implementation challenges as they require planning according to the house structure or permanent and prominent changes to the environment. For example, a sensing environment was created by placing 15 different sensors across the house of an older adult. These sensors were placed on different day-to-day appliances, such as pressure sensors on doors and motion sensors on walls [46]. Most such studies include wearables as a part of systems and were omitted from the review. Only three studies were included in this category.

##### Indirect Contact Sensor Setup

In these types of setups, a couple of sensors or sensing units are embedded inside the furniture or any other daily-use object. They require indirect contact (users to use them) to obtain the intended activity data. For example, a smart mattress in a study [67] with sensors was developed to measure the BR and HR of the person sleeping on it. The sensors were placed inside to make the system more esthetic and user friendly. Such a system can have disadvantages when daily cleaning is required, such as in older adult care homes [50]. They are also unobtrusive, but the degree of unobtrusiveness varies with user needs and context. In this review, 11 such studies were identified.

##### Sensor Setup Contributes to Implementation Outcome Acceptability

Acceptability of technology in case of older adult care is understood as the ease with which technology can be used or integrated in the day-to-day lives of older adults [4,92]. Therefore, systems that allow device-free monitoring might be more acceptable for older adult care as they can be integrated in their lives without disturbing them. Within device-free sensing, no-contact and indirect contact sensor setup were found (Table 2). Both can be acceptable, depending on the needs of older adults, use cases, among others. For example, when an older adult is sitting and watching television, a cushion that can record vital signs can be helpful; however, when they are walking, the cushion will not be helpful. In this case, a sensing system that has a no-contact sensor setup is more feasible. Within this also, it is desirable to be able to monitor at the maximum possible distances, so that only a few sensor units are sufficient for a house. Many of the included studies have tested up to the range of approximately 9 m, such as in the study by Hsu et al [53], where the walking of older adults is monitored to reflect various health issues or injuries. This study, along with external validity, conducted an acceptability study with the same participants, which showed a high rate of acceptance. This indicates that sensing systems with no-contact sensor setup tested in the range of approximately 3-9 and NLOS scenarios might be more acceptable for older adult care. However, no study other than the one by Hsu et al [53] reported conduction of acceptability testing.

#### Theme 2: Study Settings

##### Overview

The study settings encompass the type of environment used for conducting the experiments. Usually, sensor-based studies are conducted in empty rooms or laboratory setups (to observe the basic behavior of the sensors), rooms with some furniture (to validate the sensor in comparatively realistic situations), and in actual or simulated home settings (to evaluate the sensor in real-life situations). As per the observed pattern in the included studies, studies are categorized as (1) *laboratory setting with basic furniture* (including office environments and corridors) and (2) *real-life setting* (including simulation home, actual homes or apartments, and hospitals). In Table 3, studies are arranged on the basis of these two categories.

**Table 3:** Study settings.

Study settings	Included studies
Laboratory setting	[15,24-29,44,45,50,51,58,61,62,64,65,71,74-79,82,85,86]
Real-life settings	[11,18,25,46-49,52,53,55,57,59,60,68,69,72,80,81]
No information given	[54,56,63,66,67,70,73,83,84]

##### Laboratory Settings

For HAR, it is common to test the proposed systems or technology in a controlled environment before moving toward more realistic scenarios. These controlled environments are called laboratory settings. In this study, researchers tested the system with a specific experimental paradigm in a less complicated environment using healthy human participants. From such experiments, basic observations about the system or probability of using technology in HAR can be drawn, but it does not make the system compatible for implementation in real-life scenarios. For example, a high accuracy of vital signs was achieved when monitored in a controlled environment (ie, participants sitting silently very close to the device in an empty room), but as soon as the settings were changed (ie, basic furniture was introduced or distance or angle between device and participant is changed), the accuracies were negatively affected [80]. It has been observed that 50% (26/52 studies) of the studies included in this review used laboratory settings to evaluate their systems.

##### Real-life Settings

This represents the settings that are the actual use cases for the system. Specifically, in the case of device-free sensing because of the multipath propagation (the propagation of radio signals by using more than one or direct LOS path), testing in more realistic scenarios is required. Most of these systems are dependent on machine learning algorithms for data analysis, which requires a large quantity and variety of data to produce accurate results. Thus, testing the systems with more participants and in different settings increase the robustness and reliability of the system. For example, participants’ houses were used to test the radar system for monitoring sleep [52,59,69]. Using real-life settings for the evaluation of systems brings them a step closer to the implementation process. Here, 17 such studies were found.

Furthermore, it was observed that most studies that have used an indirect contact sensor setup have not provided information on study settings. This is because they require the user to use them to monitor them and are less, or not at all, affected by surroundings, unlike radio signals (device-free sensing). Of the 52 studies, 9 did not provide information on the study settings.

#### Theme 3: Age of Participants

##### Overview

This review includes USSs that were tested with adults (age: 18 years or older), including both early adults (18 years<age<55 years) and older adults (age>55 years). Among 52 studies, 2 tested older adults, 8 tested both older adults and young adults, and 21 tested their systems with early adult populations. The remaining 21 studies did not provide any information on the age of participants except mentioning that *experiments were done with adults*. Table 4 categorizes the studies based on the age groups of the participants.

**Table 4:** Age group of the participants.

Age group of the participants (years)	Included studies
>55	[46,50]
18-55	[15,25-29,45,52,54,57,58,63,65-67,73,75,77,79,84,86]
18-55 and >55	[44,47,53,59,72,76,78,85]
Adults >18 (exact information on age is missing)	[11,18,24,48,49,51,55,56,60-62,64,68-71,74,80-83]

##### Study Settings and Age of Participants Together Contribute to Implementation Outcome External Validity

External validity is a step ahead of validity in a laboratory setting. Specific to older adult care, the technology or system needs to be validated in older adult homes with older adults. Therefore, studies that have tested their systems with older adults and older adult homes can be assessed as studies with external validity.

#### Theme 4: Activities Monitored

##### Overview

As per the included studies, two major types of activities or behaviors were measured, detected, monitored, or recognized using USSs: (1) physiological states (or activities) and (2) physical activities. Only a few studies have extended work in monitoring behaviors from activities, for example, measuring sleep quality using HR and BR. Table 5 lists the studies in these two categories.

**Table 5:** Activities monitored.

Type of activities	Included studies
Physiological activities	[27,29,45,47,51,54,61,67,70,76,81,84]
Physical activities	[11,15,18,24,26,28,44,48,49,53,55-57,62,64,66,68,74,75,77-79,82,86]
Both physiological and physical activities	[25]
Behavior from physiological activities	[60,65,80,85]
Behavior from physical activities	[46,50,52,58,59,63,71,72,83]
Behavior from both	[69,73]

##### Physiological States or Activities

Within the HAR, vital signs are the most researched physiological states. By daily monitoring of vital signs, chronic illnesses (cardiovascular and respiratory disorders) can be diagnosed early [93]. This is important from the viewpoint of older adult care. Various diseases occur with age, and if they are diagnosed early, prevention can be taken on time, hence improving the quality of life of older adults. By using the USS, HR and BR were monitored. In addition, BCG signals and blood pressure were also monitored [84]. In this review, 12 studies were identified that monitored only physiological states, whereas some studies monitored behaviors from physiological states, such as vital signs to monitor cognitive load, emotional state, and sleeping behavior [60,65,80,85]. Interestingly, it can be observed that most systems used for monitoring physiological states were of an indirect contact sensing setup. This is because physiological activities such as HR and BR are movements in the range of millimeters, which is difficult to capture with wireless signals. In this review, seven studies also monitored HR and BR using a no-contact sensing setup.

##### Physical Activities

Physical activities are defined as bodily movements produced by skeletal muscles that result in energy expenditure, for example, activities of daily living (ADL; eg, walking, sitting, and eating) [92]. Similar to physiological activities, a decline in physical activity also indicates cognitive impairments and other disorders. Using USSs to monitor ADLs, various emergency situations, such as falls, can be easily tracked. Among the 52 included studies, 24 recognized or monitored physical activities, whereas nine used physical activity to monitor behaviors such as sleep, water drinking, seizure, and cognitive impairment.

##### Combination of Physical and Physiological Activities

By monitoring both these activities, more crucial and accurate behaviors can be predicted. For example, in sleep scenarios, measuring vital signs and tracking the body posture can result in a more accurate diagnosis of sleep disorders. In this review, one study explored monitoring both activities [25], whereas two studies simultaneously monitored vital signs and body movements to estimate sleep quality (behavior) [69,73].

##### Activities Monitoring Contributes to Implementation Outcome Appropriateness

In this review, the included studies monitored diverse behaviors or activities. For older adult care, it is important to monitor activities that are relevant to various stakeholders. For example, a system that can unobtrusively detect what a person is typing on a keyboard is of no use for older adult care, whereas systems that can unobtrusively monitor falls, personal hygiene, and sleep patterns are more useful for older adult care [9].

#### Theme 5: Sensing Technology Used

##### Overview

The sensing technology used in USSs can consist of different types of heterogeneous sensors. The type of sensing technology used determines the different sensor setups. Usually, for a *no-contact sensor setup*, electromagnetic or acoustic spectra are commonly used because they require a medium in which the impact of the event can be propagated. For *the indirect contact sensing setup,* various physical sensors (eg, biomedical, physical, and optical) that can transfer or translate the impact of activity can be used. Table 6 describes the sensing technology used in the included 52 studies.

**Table 6:** Sensing technology used.

Sensing technology used			Included studies
**Electromagnetic spectrum**			
	Passive infrared		[46,64,83]
	**Radio frequency**		
		Radar	[15,24-29,44,45,52,53,55,65,69,75,81,85]
		P2P^a^	[11,18,51,57-60,62,68,71,77-80,82,86]
Acoustic spectrum			[76]
**Other technologies**			
	Biomedical sensors		[63,73]
	Force sensors		[49,54,61,72]
	Thermal sensors		[50,72]
	Optical sensors		[47,67,70,84]
	Capacitive sensors		[56,74]
	Electrostatic sensors		[66]

^a^P2P: point-to-point.

##### Electromagnetic Spectrum

This technology consists of sensors that can monitor the environment and participants from a distance. Most of these systems are based on the electromagnetic spectrum (especially IR and radio waves). They can be further classified into IR- and RF-based technology:

- IR technology: it is used for short-ranged solutions (0.5-3 m) as a radar or a point-to-point (P2P) solution. Passive IR sensors are mostly used for HAR [46,64,83]. These sensors measure the IR light radiated by the objects. In this review, three studies that used passive IR were found.
- RF technology: this technology enables long-range solutions (3-9 m) and provides a higher resolution for more precise detection of small-scale human activities. This is because more fine-grained information can be collected with higher frequencies. Within RF technology, radar and P2P systems are often used. A radar system consists of one transmitter and at least one receiver at approximately the same location, making the system centralized. Often, the transmitter transmits a signal (an impulse or modulated wave), and the receiver collects different reflections of this signal. Among 52 studies, 17 such studies were found [15,18,24-29,44,45,52,53,65,69,75,81,85]. Alternatively, a P2P system can be used where the transmitters and receivers are separated in space, and thus decentralized. This review recognizes 16 studies that used P2P systems [11,18,51,57-60,62,68,71,77-80,86]. Radar-based solutions are often based on the LOS between the radar and the event or activity, whereas P2P systems are often based on the multipath propagation of a signal and are hence affected by the environment. The advantage of using a P2P system is that it can be used in NLOS environments (such as through-the-wall or behind-obstacle situations), whereas radar-based systems are often bound to direct LOS. However, radar-based systems often require less space (as they are located in a single location) and can function more easily at higher frequencies (such as mmWave), resulting in a higher resolution for HAR. Another important aspect of RF technology is the difference between higher and lower frequencies, which are frequencies in the range of RF identification and Wi-Fi (around 2.4-5 GHz), and mmWave (over 20 GHz). Here, it can be seen that for vital sign monitoring (often while sleeping or sitting still), it is more common to use higher frequencies [27,29,80], as they are more suitable for distinguishing fine-grained movements such as heartbeats. For larger activities (such as ADLs), it is more common to use lower frequencies [57] because they are more robust (can travel further) and less susceptible to noise. However, it is important to note that these are not mutually exclusive: lower frequencies (around 5-7 GHz) can still be used to monitor vital signs [26,45,60], whereas higher frequencies may still be used for general HAR or ADL.

##### Acoustic Spectrum

Acoustic waves are another way to enable truly contactless sensing. Differentiation can be made between audible acoustic waves (sound), ultrasound, and infrasound. Ultrasound is often used for distance estimation (radar-based methods). A study [76] used off-the-shelf audio speakers capable of generating up to 23 kHz (giving a limited range [18-23 kHz] to sweep over before it becomes audible to humans or requires more expensive and specialized equipment) for respiratory rate monitoring [76]. Conversely, audible sound can be captured by regular microphones, which can consist of environmental sounds (footsteps or door slamming) or vocal parameters (eg, pitch or volume). Once the actual voice is used (words and sentences), it becomes more privacy intrusive; therefore, such studies were omitted from this review.

##### Other Technologies

In this review, various studies have used different types of physical sensors such as biomedical, physical, thermal, optical, capacitive, and electrostatic sensors for HAR. Although physical sensors require contact with the subject for sensing the activities, they were made unobtrusive in the included studies by placing onto or embedding them into the infrastructure and/or objects in the environment.

- Force sensors require a physical force to register the impact on the environment (eg, vibrations for an accelerometer, applying pressure to a pressure plate, and introducing mechanical or physical stress to the stress sensor). These sensors are often placed in the environment to make them unobtrusive. Examples include pressure plates under the floor [49], geophones [54], or accelerometers on doors or windows to detect open and close events.
- Biomedical sensors require contact with the user, as they measure biological and/or chemical processes in the human body (eg, electrocardiogram and ballistocardiograph [84]). Contact is often achieved by including them in objects that participants hold close to themselves, for example, a blanket [73], pillow, or mattress [63].
- Thermal sensors change their resistance with changes in temperature, and thus can be used to monitor temperature or temperature changes. These are usually combined in heterogeneous sensor boxes [72]. In addition, the radiated temperature can be sensed by creating a thermophilic sensor, which measures the temperature difference between two points [50].
- Optical sensors work with visible light or UV emissions (luminescence sensors). An interesting application of optical sensors is in fiber-optic sensors. Light refracts and reflects differently based on the properties of the fiber (bending, temperature, and acceleration) and can therefore be used in many settings. In this review, a study [67] used a fiber-optic sensor under a mattress to measure human vital signs, whereas another study [70] used one in a headrest.
- Capacitive sensors measure changes in capacitance through capacitive coupling. In this review, one study [74] used this method by applying an electrode to the floor (transmitter) and ceiling (receiver) to measure human height. In addition, electrostatic fields exist between differently charged objects or when an object is charged differently with respect to its environment. This is often the case with the human body, as friction between the body and clothing causes the body to become electrically charged. One study [56] used an electrode on a tripod to measure the effect of capacitive coupling. Another study [66] used a piezoelectric polymer known to emit electric fields when stress is applied. This polymer was applied to the floor and used to detect different floor-impact activities (such as walking with one or more people).

##### Sensing Technology Used Contributes to Implementation Outcome Implementation Cost

Implementation cost is one of the key factors affecting the implementation process. It involves the cost of systems, efforts, and time required to install the systems [38]. Thus, to implement USSs in older adult care, studies using technologies that require minimum costs for development, deployment, and maintenance can be considered [14,94]. Technologies based on the electromagnetic spectrum require more extensive research for development, resulting in a higher cost for research and development compared with physical sensors (eg, force, biomedical, and thermal sensors). These sensors are more widely available and range in price but are often cheaper than RF-based technologies. However, these sensors are often limited in range and require multiple sensors to register events throughout a whole house setup (eg, sensors on doors or walls [46,72] or modified beds or blankets for all older adults in a care home [61,73]), and some sensors also require permanent and/or prominent structural changes to the environment (eg, implementing smart tiles [66] or adding sensors to the ceiling [46,74]), which adds additional costs to the actual implementation compared with RF-based technologies, which are often isolated boxes that offer a larger (whole house) coverage with a minimum amount of sensors [68]. For wide-scale adoption in older adult care, it is recommended to look for a solution by weighing the costs of development and deployment.

#### Theme 6: Usefulness of Unobtrusive Systems in Older Adult Care

##### Overview

The usefulness of unobtrusive sensing for HAR was obtained from the textual analysis of the included studies, especially in the context of older adult care. Out of 52 studies, 28 indicated or discussed the possible use or requirement of such a system in older adult care. These studies enlisted the various advantages of USSs for the older adults and their caregivers. From the perspective of the older adults, the included studies highlight that USSs are comfortable [18], do not require technical competency [25], are privacy aware, require less (to no) attention and compliance [72,75], are affordable [50], and can operate in NLOS situations [66,68]. From the perspective of caregivers, USSs are ubiquitous in nature [57], enable continuous monitoring [46], are easy to integrate [50], are prone to noisy environments [48], provide security [18], and are safe to use with older adults [47]. These systems are more reliable and promising for older adults affected with medical conditions such as cognitive impairment (dementia) because physicians have to rely on the caregiver’s narratives for diagnosing such conditions. For example, a study [46] aimed to detect mild cognitive impairment through an unobtrusive sensing approach to avoid delay in recognition of cognitive impairments, as it can result in severe and/or permanent damage. Similarly, another study [71] demonstrated seizure detection via wireless sensing to ensure timely intervention by caregivers to reduce the risk of injury.

In addition to these extraordinary situations, USSs are also advantageous in monitoring a wide range of general physical activities and physiological behaviors to facilitate older adult care. For example, emotion detection by methods such as FMCW radar, as demonstrated by Zhao et al [85], can help in identifying early symptoms of anxiety or depression. Accidental falls are considered as the leading cause of death in the older adult population. They not only cause physical injury but also affect physiological health. Owing to the fear of falling, most older adults limit their daily life activities, thereby impacting their quality of life. Studies [74,75] have used various USSs to ensure security by providing immediate assistance. Similarly, sleep monitoring studies [11,50,53,59,60,69,84] intended to measure the quality of sleep to promote good health by predicting sleep disorders and chronic heart diseases.

##### Usefulness of Unobtrusive Systems in Older Adult Care Contributes to Implementation Outcome Adoption

Adoption is intention, initial decision, or action to try a new technology. For older adult care, it can be seen as the intention of studies to use their systems in older adult care. Only a few of the included studies were specifically developed for older adult care. Other than these, most studies showed the intention or discussed the possible advantage of using their system for older adult care. Thus, such studies have more chances of adoption for upscaling in older adult care.

### Implementation Readiness of Identified USS Studies for Older Adult Care

The six identified factors (or themes) corresponding to the early- to midstage implementation outcomes of the framework by Proctor et al [37] were used to evaluate the implementation readiness of USSs for older adult care. They were associated with the aforementioned factors as follows: study settings and age of participants contribute to external validity, sensor setup contributes to acceptability, usefulness of USSs contributes to adoption, activities monitored contribute to appropriateness, and sensing technology used contributes to implementation cost (Table 7). On the basis of this association, the implementation readiness of the included 52 studies was checked. Among the 52 studies, studies fulfilling the associated factors were presented in the column *studies fulfilling associated factors* of Table 7. These studies can be seen as more implementation-ready than others.

**Table 7:** Implementation readiness of unobtrusive sensing system studies for older adult care.

Implementation outcomes	Identified factors and themes contributing to implementation outcomes	Studies fulfilling associated factors or themes
External validity	Study settings: studies tested in real-life settings (preferably older adult homes or at least in simulated homes) Age of participant: studies performed with older adults (Age group: 55 years or older)	[46,47,53,59,72]
Acceptability	Sensor setup: studies with no-contact sensing setups (sensors placed at a reasonable distance, approximately 3-9 m from the user)	[15,18,24-26,28,29,44,45,53,60,68,72,77,78,80,82]
Adoption	Usefulness of USSs^a^: studies that showed the possible use of their system for older adult care	[11,15,25-29,45,46,49,50,52,53,59,60,63,64,66-68,70,72-78]
Appropriateness	Activities monitored: studies monitoring activities relevant to older adult care such as life risk activities (fall) and health wellness activities (sleep)	Fall: [11,18,25,26,44,49,57,64,66,75,77,78] Sleep: [46,50,52,59-61,63,69,73,80,81,83]
Implementation cost	Sensing technology used: studies that require minimal permanent or prominent structural changes to the environment, are easy to adapt, and offer large coverage	[18,26,44,50,57,58,60,68,77,80,82]

^a^USS: unobtrusive sensing system.

It can be observed that none of the studies have considered all the factors contributing to successful implementation for use in older adult care. Although all the included studies have the potential to be used in older adult care, currently only a few studies are implementation-ready (considering some trade-offs), and most of them require improvements and tailoring to older adult care scenarios. Out of 52 studies, only five studies [46,47,53,59,72] checked external validity of their systems in real-life settings with older adults, 17 studies [15,18,24-26,28,29,44,45,53,60,68,72,77,78,80,82] used a no-contact sensor setup that can be suitable for monitoring older adults without restricting their freedom, 28 studies [11,15,25-29,45,46,49,50,52,53,59,60,63,64,66-68,70,72-78] acknowledged possible use of their system for older adult care, 24 studies (monitoring falls [11,18,25,26,44,49,57, 64,66,75,77,78] and sleep [46,50,52,59-61,63,69,73,80,81,83]) monitored activities or behavior relevant to older adult care, and 11 studies [18,26,44,50,57,58,60,68,77,80,82] used technology that requires minimal structural changes or are less expensive while implementing. As all the included studies are unobtrusive and have good accuracy, they can still be improved on the some or the other aforementioned factors for better implementation results. The study by Adib et al [44] can be considered as acceptable, appropriate, and implementation cost friendly, but it is not externally validated and was not designed considering older adult care. Similarly, for other studies, some weigh high on one outcome and less on another. Although all the identified factors are important, a trade-off depending on the use case can be made. In addition, note that for each implementation outcome, there can be more factors that can contribute to it. However, in this study, one factor was associated for each implementation outcome, which became obvious during deductive analysis.

## Discussion

### Principal Findings

This scoping review first identified 52 state-of-the-art USSs that have the potential to be used in older adult care. The deductive thematic analysis of these 52 studies helped to identify the six key factors: usefulness of USSs, types of activities monitored by USSs, type of sensing technology used to monitor activities, sensor setup used for implementing the technology, settings in which studies were tested, and the age of participants in the study. These factors in association with implementation outcomes defined by Proctor et al [37] were used to evaluate the included studies for implementation readiness. The results of this evaluation reflect that most of the included studies are at the lower end of the TRL (2/3), with only a few studies demonstrating a sufficient level of implementation readiness, thus demanding technical and behavioral research in both the pre– and post–technology implementation stages.

Furthermore, this review largely depends on the interpretation of the word *unobtrusiveness*, which is regarded as the property of sensing systems determined by the degree of attention required by the user. As per the conceptual framework developed by Hensel et al [19], the degree of attention or noticeability is categorized in eight broad dimensions which on adherence might lead to the desired *Unobtrusive* sensing system: physical, usability, privacy, functional, human interaction, self-concept, routine, and sustainability dimension [19]. Therefore, in this *Discussion* section, we aim to extend the discussion on the implications of the identified key factors in implementation readiness and unobtrusiveness by taking inspiration from the framework by Hensel et al [19].

### Sensor Setup

The sensor setup contributes to the acceptability outcome such that no-contact (NLOS) sensor setup working in the range of approximately 3-9 m or an indirect contact sensor setup can have more chances of acceptance. This is in line with the physical dimension of the conceptual framework by Hensel et al [19], which also advocates that a system is unobtrusive when it can be physically integrated into the user’s surroundings without clashing with their esthetic sensibilities. Although the degree of physical dimension may vary or a trade-off with other dimensions can be noticed, it can be accommodated by accounting user needs. For example, DeepBreath is a radar-based device for monitoring the BR by placing it near the participant’s bed in their house [81]. A similar BR device called VitalMon uses geophone sensors embedded inside the mattress [54]. Here, VitalMon is comparatively more esthetic (satisfies the physical dimension), whereas DeepBreath can work even if the user is out of the bed (satisfying the functional dimension). Similarly, for fall detection, many systems were designed and developed: SenseFall [65] used multiple sensors assembled in a box mounted on the ceiling to identify falls from other ADLs, WiVit [57] used Wi-Fi channel state information to monitor ADLs (including fall), and another system by Minvielle et al [66] embedded sensors in the floor. From the perspective of the physical dimension, the systems by Minvielle et al [66] and SenseFall are more esthetic as users cannot see anything, whereas WiVit uses at least one transmitter and receiver placed in the surroundings, requiring less structural modifications in the house.

### Study Settings and Age of Participants

For successful implementation of USSs in older adult care, the external validity of the system must be evaluated in real life or intended deployment settings with the intended age group of users. In this case, USSs should be tested preferably in the homes of older adults who usually live independently (or alone) and are vulnerable or are in the need of formal or informal care. By doing so, the functional dimension of the conceptual framework, which accounts for reliability and effectiveness, can also be satisfied. In this review, only a few studies extended the study setting from a laboratory to an in-home field study setting with seniors. For example, one study [61], monitored BR in adults (≤55 years) while they were sleeping. Upon checking the external validity of this system, it can be used for monitoring BR in older adults, as changes in BR can indicate various serious medical conditions. Although the focus of this review is limited to supporting the independent living of older adults, in real-life scenarios, various possibilities such as visits by caregivers and relatives can be anticipated. For such scenarios, one study [81] proposed an identity-matching module that used independent component analysis to identify the breathing of multiple persons, one study [58] considered leveraging the concept of Fresnel zones to determine the impact of multiple people in the surroundings, one study [54] used the Degenerate Unmixing Estimation Technique blind source algorithm to separate the heartbeat signals of multiple participants, and one study [52] demonstrated an RF-based sleep sensor to accurately monitor the sleep patterns of multiple users by combining location tracking with temporal analysis of breathing signals. Similarly, advanced data analysis can be used to separate signals from multiple persons present in the house for RF-based monitoring or no-contact sensor setup. Conversely, while using an indirect contact sensor setup, the sensing units can be embedded inside the belongings of the target user.

### Activities Monitored

A major step in developing technology for older adult care is to select the right or desired behavior or activity for monitoring. The system will be more acceptable if it measures the behaviors that are in line with the needs of stakeholders and are part of the daily routine of the older adults. From the results of a qualitative study among formal or informal caregivers of persons with dementia, it can be concluded that sensing technology should be used to monitor the risk of falls, personal hygiene, nocturnal restlessness, and eating and drinking patterns [9]. In accordance with the routine dimension of the conceptual framework, if the system is unobtrusive, it will not impact the daily routine while using such monitoring devices. Among the 52 studies, 23 (44%) focused on monitoring fall and sleep behaviors, whereas others monitored activities that can later be tailored to the older adult use case. For example, in one study [15], a human body part tracking or identification system was developed using Wi-Fi. This system can be tailored as an information provider to informal caregivers to count visitors. Similarly, other included USS studies can also contribute to older adult care after context or requirement assessment.

### Sensing Technology Used

The review reports the use of various technologies by leveraging a no-contact sensor setup to make the system unobtrusive. Among all the studies, RF-based technologies (P2P and radar) were used prominently, with more than 50% (31/52) of the studies being in that category. Within RF-based solutions, the split is quite even between radar-based and P2P-based solutions. However, other unobtrusive technologies can also be considered, which require no immediate or purposeful interaction from the participant. These sensors often need to be attached to the environment itself, such as sensor boxes [72] or smart tiles [25], or in very close proximity to the user (as an object), such as mattresses [67] and sheets [73]. Although these technologies appear promising, their development and deployment costs largely affect their implementation. Considering the conceptual framework, the sustainability dimension (affordability) and privacy dimension should be considered to make the technology unobtrusive [19]. In addition, it can be observed that researchers have succeeded in wide-range and high-resolution monitoring of human activities, enabling recognition of very small human gestures, such as tapping and picking [80], and micromovements, such as chest displacement to monitor vital signs. From the perspective of older adult care, a wide-range (or ubiquitous) and high-resolution monitoring solution can help in predicting subtle behavioral changes such as agitated behavior shown by persons with dementia without troubling them.

### Usefulness of USSs

To adopt any new technology or system, it is important to show its perceived usefulness for the relevant users. Some studies included in this review stated that their technology was designed for older adult care and thus also explained its usefulness for the same, for example, one study [46] (aimed to detect seizures), explained the adverse effect of delay in seizure detection. However, most of the included studies were on TRL 2/3 with the goal of evaluating the experimental proof of concept (explored the validity and reliability of the sensing technology in a controlled laboratory setting); hence, usefulness was not studied as a research goal. By discussing the possible use of their systems in older adult care, the intention, consideration, and initiation of the use of USSs in older adult care was shown. However, this limited knowledge of these systems has also impacted the evaluation of the effectiveness of these USSs in measuring health outcomes.

### Limitations

The review aims to enhance the implementation of the USS, specifically in older adult care. Therefore, this review has a limited scope, focusing on emerging unobtrusive technologies for older adult care from January 2011 to March 2020. Furthermore, as there is no clear consensus on the definition of unobtrusiveness, a dictionary meaning in combination with available literature was used to derive the definition of USS and UST. This variation in the understanding of *unobtrusiveness* might impact the number of identified records. The process of including studies was performed by 2 researchers (NS and JKB), but analysis of key themes obtained from the final included studies through deductive analysis was performed by 1 researcher (NS) only, which might introduce bias and impact the results and hence conclusion. However, the identified themes and their association with implementation outcomes were thoroughly discussed with other authors. Finally, although no search limitation for the type of language was used, only studies written in English were considered for final inclusion. Therefore, there is a possibility that some relevant work that was not in English is missing from the review.

### Challenges

During the review process, a number of challenges concerning implementation were encountered: (1) more than half of the included studies were not primarily designed or tested in older adult care scenarios and are early-stage experiments in laboratory settings; (2) none of the studies, except one [53], included acceptability studies along with experimental studies, and therefore, no clear picture on what users think about the systems or acceptability can be drawn; (3) the studies that targeted their systems for older adult care also require more careful consideration of factors such as testing them in older adult homes, using sensor setup that is more acceptable for older adults, or including acceptability studies; (4) for older adult care, cost is the main factor, but none of the studies provided much information on the cost associated with the system or while deploying it; and (5) the extracted geographical information indicates that most of these studies took place in nations where the required infrastructure for normalizing the use of advanced technology (such as availability of device or technology, etc) is possible. This imposes an additional challenge to normalize the use of USSs in nations where such infrastructure is not common or less idea about their cultural acceptability can be drawn.

Other than these, challenges concerning technology have also been identified. It is worth highlighting the major challenges that RF solutions can encounter in the future. One of these challenges is RF pollution: as more technologies move toward RF sensing, the amount of interference on the frequency bands increases. Two prominent bands (the 2.4 and 5 GHz) are already filled with household appliances, such as laptops and smartphones. Common ways to deal with this are multiple receivers to increase coverage or apply modulation to different transmitters and receivers to differentiate. In addition, although it is likely that RF-based sensing is a more privacy-aware solution than video-based solutions, there is an additional risk for privacy, which is the ability of RF to penetrate through walls. Although this can be used as an advantage, it also increases the privacy risk for others (eg, neighbors or guests), leading to ethical challenges. Although it is assumed that the system will be developed to promote independent living of older adults (ie, they will be staying alone mostly), there is a chance that there might be some visits from their caregivers or relatives. In addition, it is possible that RF-based systems can penetrate neighboring walls and collect data outside the household. However, these concerns can be rectified by using additional data-transferring security measures and adapting the transmitting power.

In addition, other ethical challenges involve the storage and access of collected data. Data can be stored locally for analysis through artificial intelligence algorithms (eg, neural networks), and only in emergency situations (eg, the patient falls or is feeling very unwell), a flag can be sent to the (informal) caregivers. This would be more challenging for (real-time) distant monitoring, as the actual (aggregated) data would need to be submitted. However, technologies exist that could make this as safe as possible, but there is an ongoing ethical concern about whom the data belong to.

### Future Research and Recommendations

The review shows that diverse unobtrusive technologies were explored for HAR, but most of them are still in the early stages of development, making it difficult to report implementation readiness for older adult care. Therefore, it is strongly recommended that future HAR studies intending to implement technology in older adult care should consider including implementation constructs as given by Proctor et al [37], or frameworks such as those by Greenhalgh et al [95] in advance for successful implementation. These frameworks can guide researchers in prioritizing factors (most of them identified in this review) crucial for older adult care or specific scenarios.

Challenges arise when exploring how to provide effective, safe, and meaningful personalized care while using technology. Therefore, a holistic approach must be applied that focuses on the fit between users, context, and technology. As such, it is relevant to start with user requirements, explore and identify how older adults want to live, what social and technical skills they need to be engaged in society, and use supporting technology, which then leads to the identification of values stakeholders want to achieve with products and services. Therefore, we recommend applying a holistic participatory development approach that combines value-based user-centered design, business modeling, and persuasive and positive technology. This roadmap has been applied in dementia care to develop and evaluate sensor technology and social support [96-98].

Along with using a holistic participatory development approach while developing technologies for older adult care, it is recommended to evaluate the technology or system in terms of development and implementation costs. Importantly, the developed system should be checked for external validity in older adult homes. Furthermore, RF-based solutions fit well for older adult care because of high resolution in HAR monitoring and the ease of deployment. Thus, in the future, more such solutions can be developed and implemented specifically for older adult care. In addition, experimental studies should consider adding acceptability studies as part of the research project. In this way, more meaningful insights on the perceived usefulness of the technology can be obtained from users (and perhaps other stakeholders). This results in a better adaptation to the proposed technologies. These identified factors provide the basic steps for initializing implementation from the development phase. Finally, as discussed, the importance of unobtrusiveness in eHealth, more work in defining or developing frameworks for unobtrusiveness, is desired in the future.

### Conclusions

This review is the first to explore state-of-the-art USSs suitable for older adult care. This has opened the possibilities of using existing USSs in older adult care. It shows the promising future of using RF-based technology as the USSs for HAR and its feasibility for older adult care. The assessment of identified USSs on implementation readiness is not only reflected in where improvements are required but can also be seen as guidelines for the future development of technologies.

The review also reports the points enhancing the possibility of implementation: (1) 52 unobtrusive systems that do not require direct contact with users were identified; (2) a trend in using USSs (specifically RF technology and radar-based systems) for HAR was observed, as 85% (44/52) of studies were conducted in the last 5 years; (3) among the included studies, 24 studies monitored activities or behaviors that are desired for older adult care; and (4) as for most of the studies, the primary focus was not older adult care, but they concluded or introduced how their systems can contribute to this sector. Overall, the findings of this review are intended to boost the use of USSs to provide better and on-time care to older adults and support caregivers.

All the studies included in the review are unobtrusive, but the definition of unobtrusiveness differs: some systems are very unobtrusive in physical appearance, but less unobtrusive in their implementation. The primary observation can be summed up as follows: *Unobtrusiveness or obtrusiveness is not binary; a system can have varied degrees of unobtrusiveness depending on user perspective and context*. Moreover, unobtrusive is not a quantifiable variable, but rather a qualifiable one, thus requiring a uniform and appropriate framework or instrument for informed assessment. Hence, for better understanding and fair comparisons of unobtrusiveness, a valid and reliable instrument that can be tailored to context and user attitude is required.

## Appendix

Multimedia Appendix 1 Search string for databases.

Multimedia Appendix 2 Overview of the included studies.

## Abbreviations

ADL: activities of daily living
BR: breathing rate
FMCW: frequency modulated continuous wave
HAR: human activity recognition
HR: heart rate
IR: infrared
LOS: line of sight
NASSS: nonadoption, abandonment, scale-up, spread, sustainability
NLOS: non–line of sight
P2P: point-to-point
PRISMA: Preferred Reporting Items for Systematic Reviews and Meta-Analysis
RF: radio frequency
TRL: technology readiness level
USS: unobtrusive sensing system
UST: unobtrusive sensing technology
