# Supplementary material for: Implementation of Unobtrusive Sensing Systems for Older Adult Care: Scoping Review
Source: JMIR Aging. 2021 Oct 6;4(4):e27862. doi: 10.2196/27862 (PMC8529483; doi:10.2196/27862)
Supplement: Multimedia Appendix 1 [file aging_v4i4e27862_app1.docx]

# Appendix 1

Search string for databases

| **Database** | **Search string** | **Hits found** |
| --- | --- | --- |
| Scopus | (TITLE-ABS-KEY ((unobtrusive OR nonintrusive OR non*wearable OR contactless OR wireless)) AND TITLE-ABS-KEY ( ( sens* W/3 ( system* OR technolog* ) ) ) AND TITLE-ABS-KEY ( human ) AND TITLE-ABS-KEY ( ( social* OR emotion* OR physical OR physiolog* OR health OR vital OR body ) ) AND TITLE-ABS-KEY ( ( recogni* OR detect* OR monitor* OR track* OR surveillance ) ) )  Timespan: Jan 2011- March 2020. | 1171 |
| Web of Science (WOS) | TOPIC: (unobtrusive OR nonintrusive OR non*wearable OR contactless OR wireless) AND TOPIC: (sens* AND (system* OR technolog*)) AND TOPIC: (human) AND TOPIC: (social* OR emotion* OR physical OR physiolog* OR health OR vital OR body) AND TOPIC: (recogni* OR detect* OR monitor* OR track* OR surveillance)  Timespan: Jan 2011- March 2020. Indexes: SCI-EXPANDED, SSCI, A&HCI, CPCI-S, CPCI-SSH, ESCI. | 1524 |
| ACM digital library | Title keywords & Abstract: (unobtrusive OR nonintrusive OR non*wearable OR contactless OR wireless) AND (sens* AND (system* OR technolog*)) AND (human) AND (social* OR emotion* OR physical OR physiolog* OR health OR vital OR body) AND (recogni* OR detect* OR monitor* OR track* OR surveillance)  Timespan: Jan 2011- March 2020. | 462 |
