# Supplementary material for: Implementation of Unobtrusive Sensing Systems for Older Adult Care: Scoping Review
Source: JMIR Aging. 2021 Oct 6;4(4):e27862. doi: 10.2196/27862 (PMC8529483; doi:10.2196/27862)
Supplement: Multimedia Appendix 2 [file aging_v4i4e27862_app2.pdf]

Ref.No. Authors (Year)	Study Aim	Activity (A(n))/ Behaviours monitored	Study settings	Experiment Design	No of participants	Sensing technology used	Data Analysis Methods	Main Results
[44] Adib, F. et al. (2014)	**WiTrack:** Develop a system for tracking 3D human motion.	A1. Estimate pointing gesture (3D human motion); A2. Detect fall from other PA (walk, sit on a chair and floor)	VICON motion lab	A1: Stand in different locations and perform the pointing gesture; A2: 132 experiments each PA 32 times each P	11 (Age: 22 - 56 yrs)	FMCW radar	FFT	A1: Median arm orientation error is 11.2 degrees ; A2: Precision of the fall detection 96.9% and the recall is 93.9%
[15] Adib, F. et al. (2015)	**RF-Capture:** Capture humans by tracking the 3D positions of their limbs and body parts through a wall.	A1. Body part Identification, A2. Body part tracking (left & right arm, left & right foot, and head)	Lab with basic furniture	A1: Walk towards the device, stop at a distance (in between 3-8m), and move one of the body parts. A2: Raise hand and write an English letter of choice in mid-air.	15 (Avg. Age: 31.5 yrs)	FMCW transceiver	Segmentation and pose estimation algorithms	A1: Body part classification accuracy is 99.13% at 3m and decreases with distance. A2: Median tracking error is 2.19cm and 90th percentile error is 4.84cm.
[45] Adib, F. et al.(2015)	**Vital Radio:** Monitor vital signs by using wireless technology.	A1. HR & A2. BR	Office with basic furniture.	Each P sits at 8 locations (1m away from previous location) for 2 mins facing the device (112 experiments)	14 (Avg. age: 31.4 yrs)	FMCW radar	FFT	Median accuracy at 8m for A1is 98.3% & A2 is 98.7%
[46] Akl, A. et al.(2015)	Explore the feasibility of autonomously mild cognitive impairment (MCI) detection system.	ADLs, Walking speed and number of outings/visitors	House of participant s	ADLs data was collected for 3 years while participants were staying there.	97 (Age: above 70 yrs), 18 with low MCI	PIR motion sensors, wireless contact switches	SVM, RF	MCI can be detected with an area under the ROC curve of 0.97 and an area under the precision-recall curve of 0.93.
[24] Çağlıyan, B. et al. (2015)	Explore the capabilities of the BumbleBee Radar for HAR.	PA: Walking, running and crawling	Corridors	Performe a set of 3 activities for 64 seconds at 7 different angles (between 0 and 90 degrees).	10 (Age: NA)	BumbleBee radar	k-NN	Activity classification accuracy: Walking 90%, running 88%, and crawling 93%
[47] Chen, Z. et al.(2014)	Demonstrate the feasibility of using a highly sensitive microbend multimode fiber optic sensor for vital sign detection.	A1. HR & A2. BR	MRI lab (hospital settings)	Data was collected while participants were lying in MRI machine.	11 (Age: 26-62 yrs)	Microbendi ng optical fibers	Signal Processing	Pearson correlation between mat and ground truth device for A1 is r = 0.997 and A2 is r = 0.963
[48] Chi, Z. et al.(2018)	**EAR:** Conduct fine-grained human gesture recognition using ambient RF signals generated from uncontrollable signal sources.	Gestures: Slight Kick, Pull, Wave, Swipe, Punch, Raise Arm, Circle, Stand Still, Push	In meeting room and apartment with basic furnitures	11250 sample of respective gestures were collected (less information on experiment design is available)	10 (Age: NA)	EAR receivers and various IOT devices	K -means clustering, Markov model	Gesture recognition accuracy ~92% in both environments
[49] Daher, M. et al.(2017)	Detect fall from other ADLs by using smart tiles.	Fall and ADLs (Walk, sit, stand, lie down)	INRIA-Nancy smart apartment (simulated	10 different sequences of involved activities, each sequence five times	6 (Age: NA)	Pressure sensors, 3-axis acceleromet ers	HCM (Histogram Comparison Method) in combination with SFS (Sequential	System sensitivity is greater than 90% for all postures and 94.1% for all

			smart home setting)				Forward Selection).	
[25] Diraco, G. et al.(2017)	Develop and validate a Radar Smart Sensor (RSS) to detect vital signs and ADLs.	A1. HR while doing ADLs A2. BR while doing ADLs A3. Fall (forward, backward lateral left/right) * ADLs - cooking, washing dishes, eating, sitting, sleeping	Lab with basic furniture	15 sequences of ADLs for 15 mins per P (alone and multiple people), each sequence followed by fall.	30 divided into two age groups (Avg. age: 25 & 47 yrs)	Ultra Wideband Radar	A1&A2: Empirical Mode Decomposition; A3: one-class Support Vector Machine (OCSVM) classifier	Accuracy- A1: 84%, A2: 90%; A3 sensitivity: 97% and specificity: 90%
[26] Garripoli, C. et al. (2014)	Detect fall emergencies and to localize persons by using a radar system.	Walk and fall	Lab settings with some furniture	Move/do anything without restrictions for 5 minutes followed by one simulated fall.	16 (25-39 yrs)	Radar	Least-Square SVM	100% fall detection with a maximum delay of about 316 ms
[50] Guettari, T. et al.(2016)	Design and evaluate a sensing system for estimating sleep quality.	Sleep quality	Clinical study setup	Every night sleep data (thermal signals) were collected (less information on experiment design is available)	13 Patients (Avg age: 60.84 yrs)	Thermal sensors	Self-organization map (SOM)	Deep sleep, agitated sleep and awake phase classified with 87% accuracy and 95% confidence intervals
[51] Hillyard, P. et al. (2018)	Compare the performance of four RF technologies (Wi-Fi, UWB, 1-dB quantized and sub-dB quantized RSS) for respiration detection.	BR while sleeping	Clinical study setup	8 hr BR data while sleeping.	20 Patients (Age: NA)	IR-UWB, WiFi CSI, Zigbee RSS, and sub-1 dB quantized RSS	PSD	Median error : CIR 1.32, CSI 0.60, RSS 1.56, SUB 3.36
[52] Hsu, C. Y. et al. (2017)	**EZ-Sleep:** Monitor insomnia and sleep remotely by using the radio signals.	Insomnia using Sleep latency (SL), Total sleep time (TST), Time in Bed (TIB), Sleep efficiency (SE), Wake after sleep onset (WASO)	House of participant s	100 nights of sleep data was collected.	10 (Age: 23-45 yrs)	RF-based sleep sensor	HMM	Average Error: TIB 3.15 min, TST 10.3 min, SL 4.9 min, SE 2.8 %, WASO 8.2 min
[53]Hsu, C. Y. et al.(2017)	**WiGait:** develop a radio signals based sensor to continuously measure gait velocity and stride length at home	Gait velocity and Stride length	In lab and house with basic furniture	Experimental study (Walk in the assigned place) followed by acceptability study	25 (23 - 89 yrs; 7 above 55 yrs), 18 P in lab and 14 in house	Radio sensor	FFT	Accuracy of Gait Velocity between 96.0% - 99.8% and Stride length between 88.4% - 99.3%; Acceptability study: In house participants showed higher rate of acceptance
[54] Jia, Z. et al. (2017)	**VitalMon:** Monitor vitals signs in sharing bed situations using geophones.	A1. HR & A2. BR	NA	Participants were asked to exercise (running, climbing) before collecting A1 & A2 data.	A1: 35 (Avg. age: 25.26 yrs), A2: 28 (Avg. age: 24.79)	Geophone Sensor	A1: FFT & DUET, A2: Square-law amplitude demodulation (SLD) algorithm combined with	Average error rate for A1 is 0.72 BPMhr & A2 is 2.62 BPMrr, & the median error for A1 is 1.90 BPMhr & A2 is 1.95 BPMrr.

							autocorrelation function	
[55] Kalyanaraman, A. et al. (2017)	**FormaTrack:** Use body shape as weak biometric for differentiating people.	Body Shape based on features such as head size, shoulder size, or torso size.	House of participants	Each participant walked for 7 days, 25 times in one session.	8 (Age: NA)	IR-UWB transceiver chip	FFT followed by SVM classifier	Tracking precision 100%, recall 99.86%, direction 99.7% and identity 90.3%
[18] Klein Brinke, J. et al. (2019)	Validate the strength of COTS WiFi devices in HAR	PA: Clap, walk, wave, jump, sit, fall	Apartment of participants	Perform the same activity for 250 seconds (50 trials of 5 seconds).	6 (Age: NA)	Tx-Rx system	CNN	Single-participant activity validation up to 93% and cross-validation 30%-60%
[27] Kuutti, J. et al.(2015)	Evaluate a doppler radar for detection of vital signs with radar-signal reflecting aluminium-coated surfaces.	A1. HR & A2. BR	Test room constructed of thick insulation boards and aluminum coating.	Lay in 4 positions (supine, prone, right and left side) and 2 sitting positions (under & away from radar) for 2 mins while breathing normally followed by holding breath.	10 (Avg age: 33yrs)	Microwave Doppler radar sensor	Signal Processing	Correlation with ground truth respiration signal is 0.84 and 0.93 for normal breathing and breath hold. (Less information available)
[56] Li, M et al.(2018)	Design and validate a gait monitoring system using electrostatic sensing.	Walk slowly, fast and at a normal pace.	NA	Walk slow, normal and fast pace for at least 20 seconds per pace, 300 valid gait cycle data samples were collected.	10 (Age: NA)	Electrostatic field sensing measurement	Thresholding (foot pressure), mathematical model validation for electrostatic field sensing (EFS)	Gait monitoring accuracy: 97%
[57] Li, X. et al.(2018)	**WiVit:** Design a vitality monitoring system (detect motion and location) using Wi-Fi devices.	A1.Walk, A2.PA: Run, Sit down, and Fall	Empty room, office room with basic furniture, and a smart home	Walk freely or keep still and do PA; 3427 instances of PA were collected.	5 (Avg age: 26 yrs)	Wi-Fi cards as the transmitter and receivers.	HMM	Precision of activity detection for A2 98% and FNR is 1%, for A1 96% and FNR is 5% in smart homes.
[58] Lin, W. et al.(2018)	**DW-Health:** Recognize daily activity of drinking water.	A1. Drinking behaviour, A2. Drinking-like behaviour (jumping, pushing glasses), A3. Non-Drinking behaviour (keystroking, and moving mouse), A4. Water intake A5. Body state (calm or not clam)	Empty room and office with basic furniture	A1 A2 and A3 at 6 different locations. A5: calm state followed by different sports for non calm state	6 (Age: 21-38 yrs)	Wi-Fi Tx (2 antennas) and Rx system (3 antennas)	Variational mode decomposition (V DM) and Principal component analysis (PCA)	Average detection rate- A1: 97% , A2: 89.01%, A3: 84.83% A4: No accurate results , A5: 90%
[59] Liu, C. et al.(2019)	**TagSleep:** Detect sleep sound-activities by using RFID tags.	A1. Sleep Posture, A2. Sleep sound activity (snore, cough and somniloquy)	House of participants	Two months of sleep data was collected by placing tags to the nearby wall and antenna on the nightstand.	30 (Age: 18-60 yrs)	3 RFID tags and 1 RFID reader	RF	Precision, recall, accuracy for A1 is ~98% for A2 is ~96%

[60] Liu, J. et al.(2018)	Track vital signs during sleep by using off-the-shelf WiFi.	A1. HR, A2. BR, A3. Sleep	Lab (for A3) and two apartment's bedrooms (for A1 and A2) with basic furniture.	Three-months of data for A1 & A2. For A3, lie on bed in four sleep postures (prone, supine, curl-up and recumbent), 40 mins each.	6 (Age: NA)	3 sets of Wi-Fi router as Tx and PC as Rx	A1 & A2: power spectral density (PSD); A3:DA, SVM, kNN, and RF	A1: 90% of estimation errors are less than 4bpm; A2: 80% estimation errors are less than 0.5bpm; Accuracy of A3 by DA, SVM > 80% , & by kNN & RF >90%
[61] Liu, J. J et al.(2014)	**BreatheSense:** Evaluate the effectiveness of the torso localization algorithm for respiratory signal extraction.	BR while sleeping	Lab settings with some furniture	Sleep in any preferred style and some experiments were done in tilted bed position as well.	12 (Age: NA)	High-density pressure sensor array	Pressure images analysis (torso localization)	Torso area shows 1.7% error w.r.t. to ground truth
[62] Liu, C. et al. (2019)	**Wi-CR:** Develop a human action counting and recognition system using Wi-Fi signals.	A1. Start and end times of actions (Walk & Squat), A2. Number of actions	Lab settings with some furniture	Experiments were conducted in the middle of the LOS having 1.5 seconds gap between two actions (walk and squat) and each action for 50 times.	5 (Age: NA)	WiFi router as a Tx, and a PC as the Rx	DWT based waveform feature extraction method and DTW and KNN for action recognition	Accuracy: 95% for action counting and an 90% for action recognition rate
[63] Liu, Y. et al. (2015)	**WihzPAD:** Develop a Bed-Centred Telehealth System (BCTS), to collect health data from beds for homes and nursing homes.	Sleep on bed (flat, turn left, turn right) then get off the bed	NA	Each position for 30 seconds and repeated 3 times for each P.	15 (Age: 20-30 yrs)	Motion sensors	Mathematical analysis	Sensitivity: on-bed & off-bed detection is 1.00; the lying flat and side detection in are 0.79 and 0.92
[64] Luo, X. et al. (2012)	**SensFall:** Design and implement fall detection system	Fall followed by ADLs (Sit down, stand up from a chair, walk and jog)	Office with basic furniture.	Fall followed by ADLs ten times. 80 fall samples and 320 normal activity samples were obtained.	8 (Age: NA)	7 PIR sensors assembled in a box	HMM	Fall detection rate 86.5%
[65] Matkovic, T. et al. (2018)	**Wi-Mind:** Evaluate wireless system for cognitive load inference.	8 cognitive tasks of different levels (like finding hidden patterns) to elicit cognitive load.	Office with basic furniture.	Each of the 8 tasks were presented three times having three different difficulty levels. Data from Wi-mind is collected for appx. 45 minutes.	23 (Age: 20-38)	Software defined radio based (SDR) radar	FFT followed by RF, NB and SVM	Cognitive load detection accuracy by RF - 83%, SVM - 54%, NB -77%
[66] Minvielle, L. et al. (2017)	Design a fall detection system by embedding sensors in the floor.	ADLs: Walks with one or more persons, movements with a chair, sitting, jumping, running, picking objects, followed by falls.	NA	Fall followed by other respective ADLs.	28 (25-45 yrs)	Piezoelectric sensor	RF	Maximum fall detection accuracy with RF was 98.4%
[67] Podbreznik, P. et al. (2013)	Develop a human vital sign monitoring system using cost-efficient plastic optical fiber (POF)	A1. HR & A2. BR	NA	Lay in supine on the mattress for 2 mins.	10 (Avg. age: 31.8 yrs)	Plastic optical fiber (POF) system	Spectral Analysis	Precision for A1 98.8% & A2 97.9%; Sensitivity for A1 99.4 & A2 95.3%

[68] Qifan, P. et al.(2013)	**WiSee:** Develop a whole-home sensing and human gestures recognition system using wireless signals.	PA: Push, dodge, Strike, Pull, Drag, Kick, Circle, Punch, Bowling.	Office building and a two-bedroom apartment	Perform a set of respective PA in LOS, NLOS and through the wall. (Less information available)	5 (Age: NA)	SDR radio with multiple Tx (single antenna) and multiple Rx (5 antennas)	Classification based on sequence recognition of the activity in the doppler shift	Recognition accuracy: 94% on average (SD: 4.6%)
[69] Rahman, T. et al. (2015)	**DoppleSleep:** Develop a sleep sensing system for tracking sleep quality using COTS radar modules.	A1: HR, A2: BR, A3: Body movements while sleeping.	House of participant s	110 hours of sleep data from 16 sleep sessions.	8 (Age: NA)	K-band radar module	A1&A2: Mean Absolute Error (MAE) & Normalized Root-Mean-Square Deviation (NMRSD), A3:Root mean square, B1: Random forest	Overall mean absolute error A1: 1.98 & A2: 3.29 cycles pm. B1: sleep or wake event with a recall of 89.6% and sleep stages with a recall of 80.2%.
[70] Sadek, I. et al.(2017)	Measure interbeat intervals by using a microbend fiber optic sensor.	HR while doing various activities.	NA	During the data acquisition Ps answered questionnaires, took rest (no movement), and had stress relief massage.	50 (Age: NA)	Microbend fiber optic sensor	Maximal overlap DWT	Mean Avg Error for the MODWT (7.31 +-1.60)
[28] Seifert, A-K. et al. (2019)	Develop and validate a micro-doppler radar system for in-home gait analysis.	Normal walk, walk while limping one leg or both legs, use cane	Office with basic furniture.	Walk slowly toward and away from the radar system doing each activity one by one.	10 (Age: 23 ± 2.6 yrs), 4 Patients (Age: NA)	UWB Radar	Model verification and thresholding	Gait avg. accuracy: 93.8% & cross-validation 80%
[71] Shah, S.A. et al.(2018)	Develop and validate a seizure episode detection system.	Press-ups, walk, sit on a chair, squats followed by simulated epileptic seizure	Lab (with simulation of home/hos pital settings)	Each experiment ~250s, time per activity varied (press-ups 50s, walking 100s, sitting on chair 25s, squatting 25s, epileptic seizure 25s).	10 (Age: NA)	S Band Tx-Rx Channel Input Response (CIR)	SVM, k-NN, RF	Seizure episode detection accuracy: ~90%
[72] Stucki, R.A. et al. (2014)	Evaluate assistive technology that recognizes and classifies ADLs.	8 ADLs : Sleeping, Grooming Toileting, Getting ready for bed, Cooking, Eating, Watching TV, Seated activity.	House of participant s	Activities were recorded for 20 days.	10 (Age: 28-79 years), 1 84 yrs	Temperatur e, humidity, luminescenc e, motion and acceleration sensors assembled in the box	Classifier for classifying ADLs was developed.	System reliability: 99.53% , Sensitivity: 91.27%, and Specificity 92.52%
[29] Sun, G. et al.(2014)	**Vital-Cube:** Develop a vital sign monitoring system microwave radar.	A1. HR & A2. BR	Lab with basic furniture	Participants were asked to sit in front of the device. (Less information available)	16 (Avg Age: 23 ± 3yrs)	Microwave radar	Bland-Altman Analysis	A1: correlation with ECG , r = 0.87; A2: correlation with respiratory belt, r = 0.91
[73] Takano, M. et al.(2018)	Develop and validate a fabric-sheet sensing solution to monitor in-bed physiological and behavioral signals.	A1. BR, A2. Different sleep postures	NA	Monitored for A1, A2 (change posture every 20 seconds); Sleep & BR data for 6hrs each.	7 (Age: 22–45 yrs), 2 (Age: 24yrs), 7 (Age: 20–23 yrs)	Fabric-sheet unified	K-means clustering	Classification accuracy A1: ~80% & A2: ~88%

						sensing electrode		
[74] Valtonen, M. et al. (2011)	Design a human height and posture measurement system using capacitive sensors.	Height & posture	Lab with basic furniture	Stand, crouch with heels lifting off the ground, and crouch with heels staying on the ground.	14 (Age: NA)	Multiple Rx and Tx were used to create capitative sensors	Mathematical analysis	Height of a person can be measured with 5.2 cm and 14.3 accuracy in standing and all other postures
[75] Wang, K. et al.(2019)	Demonstrate the potential of detecting falling events using commodity mmWave sensor.	Various fall activities : Slip, fall from chair, lose conscious, losing balance, sit, slight forward and backward lean, bending down, random wandering	In corridor, offices and tea/ coffee room with basic furniture	Experiment paradigm follows various fall activities.	28 (Age: 20-45 yrs)	mmWave sensor	Power-based method for body-height estimation and an OBB-based method for body-orientation approximation	Accuracy: 88.79%, Precision: 75.20%, Recall: 92.16% and F1 score: 82.82%,
[76] Wang, T. et al. (2018)	Develop and validate a FMCW acoustic radar to monitor respiratory rate.	BR while sleeping	Four rooms of varied size (less informatio n available)	BR was monitored for 200 seconds in three different postures.	7 old age , 13 (Age: NA)	Speaker and microphone as transceiver	Cross-correlation FMCW	Error lower than 0.35 breaths/min under 0.7m
[77] Wang, W. et al. (2017)	Develop and validate a system to detect HAR using COTS WiFi devices and CSI.	Walk, fall, sit, open refrigerator, run, fall, boxing, pushing one hand, brushing teeth	Lab with basic furniture	Perform activities at different locations w.r.t. Tx-Rx, a total of 1400 samples were collected.	25 (Age: 19-22 yrs)	Tx-Rx system	PCA followed by HMM	HAR accuracy: 96%
[78] Wang, Y. et al. (2018)	Develop and validate an RFID reflection based HAR system.	PA: Stand, sit, raise hand, drop hand, walk, fall, rotate, get-up	3 office rooms with basic furniture	Sets of PA were performed, each activity repeating for 240 times by each P in 3 locations.	12 (Age: 24-58 yrs)	Antenna and RFID tags	Segmentation algorithm followed by DT, SVM. RF, NB, QDA)	Avg. precision HAR by RF: 93.5%
[79] Wenyuan, L. et al. (2018)	**WiCoach**: Develop a system that uses different parts and movements of the human body for activities recognition.	Eight section brocade (fitness composed of eight sections of movements involving hands, arms, head, neck, torso, legs, ankles, feet)	Empty hall room and office with basic furniture.	Perform ESB in LOS and NLOS (0.5m away)	6 (Avg. age: 29 yrs)	Wi-Fi router as Tx and PC as Rx	DTW followed by k-NN	Average classification accuracy of the ESB was 0.92 with standard deviation 0.07.
[80] Yang, Z. et al.(2017)	Design a vital sign and sleep monitoring systems using mmWave sensors.	A1. HR, A2. BR, A3. HR & BR while sleeping	Lab and apartment bedroom with basic furniture	A1 & A2: Sitting at three different incident angles for 10 mins; A3: Sleep for 3 minutes (repeated 10 times) in four different postures	7 (Age: NA)	mmWave Tx and Rx	Less information available	A1: 0.5 Bpm (error), A2: 2.5 bpm (error), A3 accuracy : ~98% (BR) & ~95% (HR)
[11] Yao, L. et al.(2017)	Develop and validate an RFID RSSI HAR system.	PA: Sit, stand straight, sit-stand, walk, high arm waving (3 versions), kick, bend over, crouch, and fall	Office and house with basic furniture.	Perform sets of PA,120 seconds each.	6 (Age: NA)	Antenna and RFID tags on the wall	Activity Dictionary Learning algorithm	HAR Accuracy: 80% with 10% training, 90% with 20% and 50-66% for cross validation

[81] Yue, S. et al.(2018)	**DeepBreath:** Disentangle multi-person radio frequency breathing signals.	BR while sleeping	House of participant s	Couples were asked to sleep for 21 nights, collecting over 150 hours of sleep data.	13 couples (Age: NA)	FMCW radar	Independent component analysis (ICA)	Average error 0.140 breaths per minute
[82] Zhang, O. et al.(2016)	**Mudra:** Develop fine-grained finger gesture recognition.	A1. Finger movements (shoot, pick, come, tap, double pick, double tap, circle, twist, go)	Office with basic furniture.	Different angles and distances between Tx and Rx was tested for A1. For tapping 24 hours data was collected by placing Tx and Rx on the office table.	5 (NA)	WiFi adapters with two Rx and one Tx antennas	Stretch limited DTW (amplitude thresholding)	Gesture recognition accuracy: at 2cm 98%, and 96% for 0.5-7m
[83] Zhang, S. et al. (2018)	Develop and validate a sleep monitoring system using pyroelectric infrared sensing.	Sleep pose recognition: wave left, right & both hands, raise left, right & both hand, raise left leg & right leg, Roll left & right	NA	Ps were asked to repeat each action 10 times at a normal rate (100 samples of each action for training) for 5 seconds each.	10 (Age: NA)	Passive Infrared sensors	k-NN	Sleep pose recognition avg. accuracy: 83.55%
[84] Zhang, Y. et al.(2019)	Demonstrate continuous BCG based human identification system using a microbend fiber sensor.	Ballistocardiograph (BCG)	NA	Data for 80 sec and 360 sec were collected from 15P and 30P when they were sitting on a chair by placing a smart cushion.	15 & 30 (Avg Age: 24 ± 5 yrs)	Microbend fiber sensor	1D-CNN	BCG accuracy 100% for 15P group, and 90% for 30P group
[85] Zhao, M. et al.(2016)	**EQ-Radio:** Develop and evaluate the feasibility of emotion recognition using radio frequency signals.	A1. HR, A2. Emotions: Joy, Pleasure, sadness, anger.	5 different office rooms with basic furniture	A1: 130,000 Heart beats were collected; A2: Stimuli were used to elicit the emotions and100 signal sequences for each emotion was collected.	A1: 30 (Age: 19-77yrs); A2: 12 (Age: NA)	FMCW radar	A1: Mathematical Analysis; A2: Person-dependent and independent classifier.	A1: Error less than 2%, A2: Average accuracy of person-dependent emotion classification 87.0%. & person-independent classification 72.3%
[86] Zhu, Y. et al.(2019)	Develop and validate a virtual fitness assistant based on CSI.	A1. Workout detection, A2. User identification	Lab with basic furniture	7 typical free-weight exercises (3 - 6 sets, 5 - 40 repetitions)	10 (Age: 22-30 yrs)	WiFi Rx-Tx	Convolutional neural networks	Avg. accuracies A1: 97%, A2: 98%

Multimedia appendix 1: Overview of 52 included studies

Abbreviations used in table: ADL: Activities of daily living, BR: Breathing Rate, BCG: Ballistocardiogram, COTS: commodity off-the-shelf, CDF: Cumulative distribution function, CIR: Channel impulse response , CNN: Convolutional neural networks, CSI: Channel state information, DA: Discriminant Analysis, DTW: Dynamic time wrapping, DUET: Degenerate unmixing estimation technique, DWT: Discrete Wavelet Transformation, FFT: Fast Fourier Transform, FMCW: Frequency-Modulated Continuous Wave, IR-UWB: Impulse radio Ultra-wideband, HAR: Human activity recognition, HCM: Histogram Comparison Method, HMM: Hidden Markov model, HR: Heart Rate, KNN: K-nearest neighbour, MAE: Mean Absolute Error, NA: not available, NMRSD: Normalized Root-Mean-Square Deviation, P: Participants, PA: Physical Activities, PSD: power spectral density, QDA: Quadratic Discriminant Analysis, RF: Random Forest, RFID: Radio frequency identification, ROC curve: receiver operating characteristic curve, RSS: Received signal strength, Rx: Receiver, SDR: Software defined radio-based radar, SFS: Sequential Forward Selection, SOM: self-organization map, SLD: Square-law amplitude demodulation, SVM: Support vector machine, Tx: Transmitter, UWB: Ultra-wideband
